# Training-induced impairment of endothelial function in track and field female athletes

**DOI:** 10.1038/s41598-023-30165-2

**Published:** 2023-03-01

**Authors:** Marcin Grandys, Joanna Majerczak, Marzena Frolow, Stefan Chlopicki, Jerzy A. Zoladz

**Affiliations:** 1 Chair of Exercise Physiology and Muscle Bioenergetics, Faculty of Health Sciences, Jagiellonian University Medical College, Ul. Skawinska 8, 31-066 Krakow, Poland; 2 Jagiellonian Centre for Experimental Therapeutics (JCET), Jagiellonian University, Krakow, Poland; 3 Department of Experimental Pharmacology, Chair of Pharmacology, Jagiellonian University Medical College, Krakow, Poland

**Keywords:** Endocrinology, Physiology, Cardiovascular biology

## Abstract

Professional athletes are often exposed to high training loads that may lead to overfatigue, overreaching and overtraining that might have a detrimental effects on vascular health. We determined the effects of high training stress on endothelial function assessed by the flow-mediated dilation (FMD) and markers of glycocalyx shedding. Vascular examination as well as broad biochemical, hormonal and cardiometabolic evaluation of sprint and middle-distance female runners were performed after 2 months of preparatory training period and compared to age-matched control group of women. Female athletes presented with significantly reduced FMD (*p* < 0.01) and higher basal serum concentrations of hyaluronan (HA) and syndecan-1 (SDC-1) (*p* < 0.05 and *p* < 0.001, respectively), that was accompanied by significantly lower basal serum testosterone (T) and free testosterone (fT) concentrations (*p* < 0.05) and higher cortisol (C) concentration (*p* < 0.05). It resulted in significantly lower T/C and fT/C ratios in athletes when compared to controls (*p* < 0.01). Moreover, fT/C ratio were significantly positively correlated to FMD and negatively to HA concentrations in all studied women. Accordingly, the training load was significantly negatively correlated with T/C, fT/C and FMD and positively with the concentrations of HA and SDC-1. We concluded that young female track and field athletes subjected to physical training developed impairment of endothelial function that was associated with anabolic-catabolic hormone balance disturbances. Given that training-induced impairment of endothelial function may have a detrimental effects on vascular health, endothelial status should be regularly monitored in the time-course of training process to minimalize vascular health-risk in athletes.

## Introduction

Aiming to achieve the best performance, the athletes are often imposed to a very high training workload which exceeds their adaptive potential (see e.g.^1^). This may lead to an imbalance between training stress and adequate recovery, resulting in overfatigue, overreaching and overtraining^2^. It was suggested that the best strategy to identify overloaded athletes is the regular monitoring of a different training-related variables (i.e. biochemical, hormonal, cardiac, psychological) that are accompanied by unexplainable decrease in performance^2^.

One of the most often used hormonal marker of the excessive training loads is testosterone (T) or free testosterone (fT) and their relation to cortisol (C)^3^—so called anabolic-catabolic hormone balance. It is interesting that heavy training periods often led to decrease in basal T and fT concentrations and as a result, to reduced T/C and fT/C ratios (see e.g.^3,4^), similarly to the changes observed in normal aging^5,6^. At the same time, impaired androgen profile in aged men has been recently viewed as an important factor in aged-related vascular dysfunction^7^ and it was demonstrated that androgen deprivation therapy has a detrimental effects on vascular health in cancer patients^8^. In general it is accepted and there is overwhelming evidence that exercise training have a beneficial effect on vascular system^9^, however transient post-exercise deterioration of endothelial function was demonstrated in humans^10^ as well as in experimental animals^11^.

**Figure 1:**
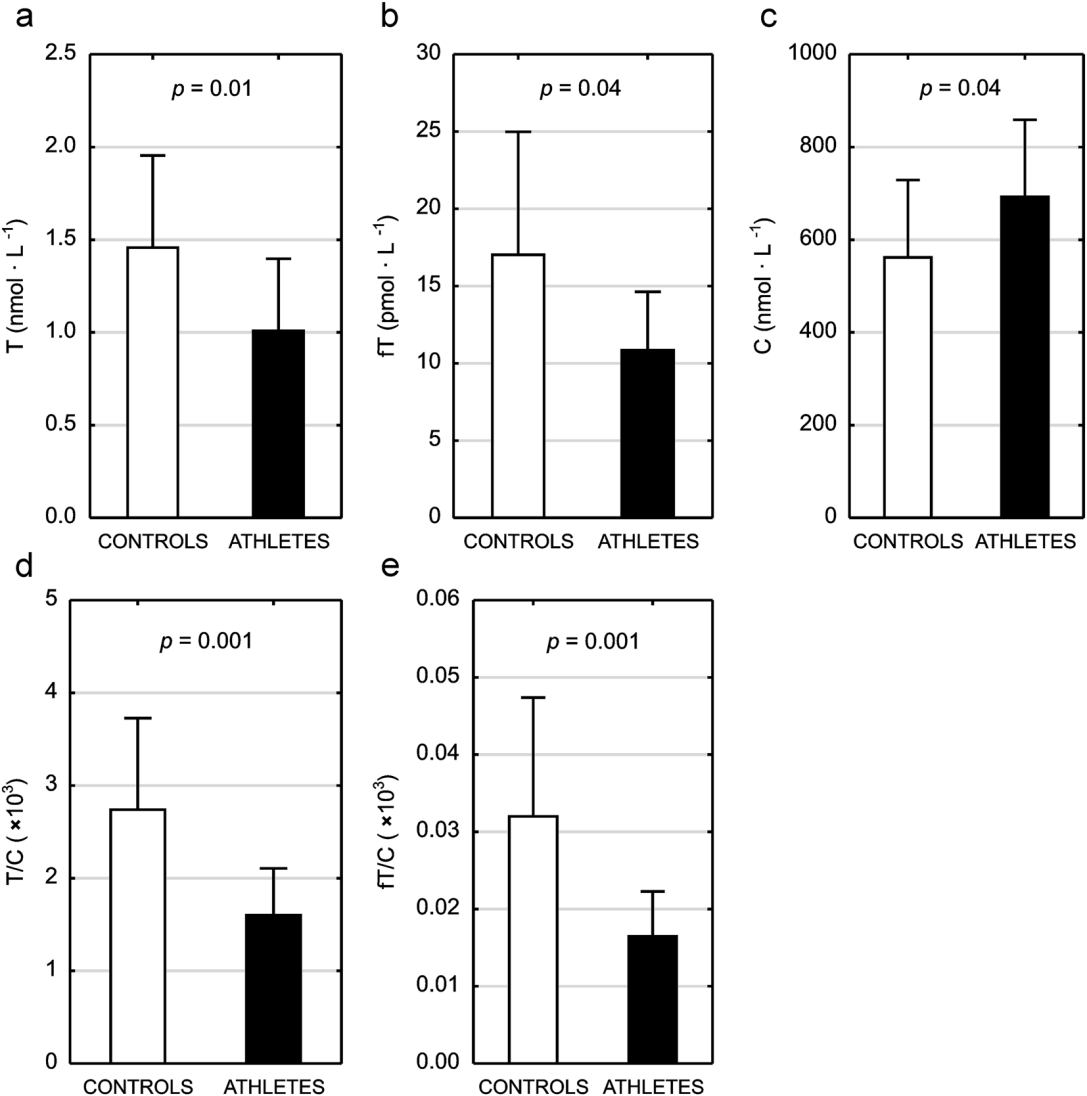
Androgen status in female athletes and age-matched control group. Testosterone (T—panel **a**); free testosterone (fT—panel **b**); cortisol (C—panel **c**); testosterone to cortisol ratio (T/C—panel **d**); free testosterone to cortisol ratio (fT/C—panel **e**).

Indeed, decreased flow-mediated dilation (FMD)—an index of endothelial function—occurs immediately after single exercise session, but it was usually temporary with later (supra)normalization response that occurs 1–24 h postexercise, accordingly to the biphasic pattern of FMD changes after an acute bout of exercise (see Fig. 1 in^12^). Similarly in mice, post-exercise impairment of endothelial function was transient with maximum changes 2 h after completion of exercise and an acute bout of exhaustive running resulted in decreased production of NO^•^ with concomitant increased production of superoxide (^**·**^O_2_^−^) without changes in thrombogenicity^11^. It suggests that adaptive and transient post-exercise endothelial dysfunction result in temporary reduction in NO^**·**^ bioavailability that however does not modify thromboresistance in healthy mice after exercise. Furthermore, adaptive nature of endothelial response to exercise was also shown in female ApoE/LDLR^−/−^ mice with early and advanced genetically-driven hyperlipidemia and atherosclerosis^13^. ApoE/LDLR^−/−^ mice displayed a clear-cut overactivation of platelets, as compared with female WT mice, but quite surprisingly, platelet activation in ApoE/LDLR^−/−^ mice was attenuated post-exercise. These mechanistical studies in mice underscore transient and adaptive nature of post-exercise endothelial dysfunction after short-term exercise.

Nevertheless, there is also evidence that after strenuous exercise (a marathon run) a decreased FMD was not restored after about 24 h postexercise^14^. Moreover, it was demonstrated that high contractile activity during exercise may lead to both high mean blood pressures and large retrograde flow and share rate that were related to impaired endothelial function (see respectively^15,16^). One may speculate that repetitive exhaustive exercise performed without sufficient rest can lead to chronic vascular dysfunction manifested among others in decreased FMD. Nevertheless, FMD response to exercise training in elite athletes (especially in females) was rarely studied and the results are not so clear as in the case of healthy untrained or diseased individuals^17^.

Our previous results^18^ confirmed some reports that the athletes have similar FMD values as the untrained subjects^19,20^, but contradicted other studies that found higher^21^ or lower FMD^17^ in elite or sub-elite athlete groups. Knowing the fact that the training-induced changes in resting FMD are dependent upon single exercise-induced changes in FMD^22^ and that post-exercise decrease in FMD occurs in an intensity-dependent manner^23^, we have hypothesized that resting values of brachial artery flow-mediated dilation and endothelial glycocalyx shedding may be dependent upon the magnitude of the training stress applied in varied periods of athletes yearly training. To verify this assumption, we have examined brachial artery flow-mediated dilation (FMD) as well as markers of glycocalyx shedding and performed broad biochemical, hormonal and cardiometabolic evaluation of professionally trained female athletes directly after 2 months of preparatory period in the yearly training program and compared them to age-matched, healthy women.

## Methods

### Subjects

Female athletes (ATH, n = 15) and age-matched control women (CON, n = 15) participated in this study. The mean (± standard deviation, SD) age, height, body mass and body mass index were comparable in both groups (see “Results” and Table 1). All participants underwent a standard medical evaluation and a routine blood tests to check for any potential contraindications for the study participation. To be included in the investigation, female were required to be healthy, non-smoking, free from cardiovascular and other diseases, non-pregnant, free from medications and without amenorrhea. Although we made an effort to recruit women with regular menstrual cycles for the study, some of the female athletes (n = 4) had irregular menstrual bleeding (shortest to longest cycle variation ≥ 8–10 days), accordingly to the latest system of the International Federation of Gynecology and Obstetrics (FIGO) for nomenclature of symptoms of normal and abnormal uterine bleeding in the reproductive years (please see^24^). We decided to involve the athletes with irregular menstrual bleeding to have sufficiently high sample size of athletes at desired national and subnational level. Since the phenomena of abnormal uterine bleeding is commonly reported in the female athletes (e.g.^25^), we consider that our studied group of athletes represents a typical sample of the female athletes. Moreover, in this 4 female athletes we found that mean menstrual cycle length (within the time-frame of the study) was 30.3 ± 5.9 days and it was not markedly different from the rest of the ATH group (28.5 ± 1.9, n = 11). The basic anthropometric, cardiovascular, hematological and blood biochemical characteristics of athletes and age-matched control group is given in Table 1.

**Table 1:** Basic anthropometric, cardiovascular, hematological and serum biochemical parameters of the controls (CON, n = 15) *vs* female athletes (ATH, n = 15).

	CON	ATH	*p *value
Anthropometric data			
Age, year	22.7 ± 1.0	23.7 ± 3.3	0.51
Height, cm	165.2 ± 4.4	169.7 ± 3.8	0.02
Body mass, kg	57.7 ± 8.3	58.8 ± 5.9	0.62
Fat mass, %	23.6 ± 7.6	21.3 ± 5.4	0.37
BMI, kg m^−2^	21.1 ± 2.8	20.4 ± 1.3	0.46
Cardiovascular data			
cSP, mmHg	120 ± 11	119 ± 7	0.76
cDP, mmHg	68 ± 9	64 ± 5	0.16
PP, mmHg	52 ± 5	55 ± 7	0.20
HR, bt min^−1^	71 ± 12	54 ± 8	0.0001
Hematological and blood biochemical data			
Hct, %	41.5 ± 2.4	41.3 ± 1.7	0.97
Hb, g dL^−1^	13.4 ± 1.0	13.6 ± 0.7	0.68
E, × 10^12^ L^−1^	4.59 ± 0.36	4.52 ± 0.20	0.74
L, × 10^9^ L^−1^	6.21 ± 1.60	5.64 ± 1.23	0.45
PLT, × 10^9^ L^−1^	283 ± 65	287 ± 60	0.81
Na^+^, mmol L^−1^	138.9 ± 1.2	138.1 ± 1.5	0.10
K^+^, mmol L^−1^	4.11 ± 0.27	4.08 ± 0.21	0.93
Cr, μmol L^−1^	67.5 ± 5.4	76.2 ± 11.9	0.01
Alb, g L^−1^	41.0 ± 2.2	42.0 ± 1.6	0.17
SHBG, nmol L^−1^	55.7 ± 25.3	68.8 ± 35.4	0.15

Data are given as mean ± SD. *BMI* body mass index, *cSP* central systolic blood pressure, *cDP* central diastolic blood pressure, *PP* pulse pressure, *HR* heart rate, *Hct* hematocrit value, *Hb* hemoglobin concentration, *E* erythrocyte count, *L* leukocyte count, *PLT* platelet count, *Na*^+^ sodium concentration, *K*^*+*^ potassium concentration, *Cr* creatinine concentration, *Alb* albumin concentration, *SHBG* sex hormone-binding globulin concentration.

Female athletes belonged to the group of track and field runners competing at national and subnational level in sprint-to-middle distance runs (12 of them specialized in 100–400-m sprints and three of them were 800–1500-m runners) and they had on average 9.3 ± 2.9 years of training background. At the moment of testing (blood and vascular testing), that took place between the end of December and mid-January, the athletes were at the end of the first preparatory phase of training, about one and half month before the main event of the indoor season—National Athletics Indoor Championship. This phase of training can be characterized as a general preparation period starting with gradually increasing training workload through low to moderate intensity and high volume conditioning activities and ending with more sport-specific training of higher intensities and moderate to high volumes. We included in this study both sprinters and middle-distance female runners in order to have sufficiently high sample size of athletes at desired national and subnational level. Although, the training content is different for both specializations, the preparatory period of the season involve high training volume in both sporting events. This period of training is characterized in both sprinters and middle-distance runners by an increase of aerobic training sessions in form of running at moderate/heavy exercise intensities and is much more demanding than that conducted in the transition period between the end of competitions and the onset of the preparation period. The similarity between the two groups can be also illustrated by the fact that middle-distance runners, in order to compete successfully, also need to do a significant amount of sprint training aimed at increasing their ability to run 400-m fast (allowing them to sustain high speed in middle distances) which actually is a sprinting distance. Therefore, both short- and middle-distances female runners were eligible to participate in the study, which aimed to determine the effect of training stress on endothelial function.

Based on a general survey on sports performance, physical activity and diet conducted at the enrollment stage of the study, athletes declared that they had on average 12.9 ± 2.4 h of training per week during their last year of training, and they stated that similar amount of training load was also maintained during the last 2 months before entering the study. On the other hand, CON group were not involved in any regular physical conditioning program and had on average 2.7 ± 1.2 h of spontaneous physical activity per week. In terms of the participants’ diet, it appeared that all of them had a similar dietary pattern. It was characterized by low-fat, high-fiber, high-carbohydrate, dairy-rich diet, with moderate meat consumption.

### Experimental testing

During the first testing day in the laboratory, blood samples were taken at rest from the antecubital vein, after overnight fasting between 7:30 and 8:30 a.m. from both CON and ATH group. The vascular examination (second testing day) for each participants was also performed in the morning hours (7:30–10:00 a.m.) in the fasted state at least 48 h after blood testing and on average within 7 days from this first visit in the laboratory. For both blood and vascular examination, the participants were instructed to refrain from caffeine, alcohol and energetics drinks 24 h before the testing and they were also asked not to perform strenuous exercise within this time frame. To ensure similar measurement condition with respect to the phase of the menstrual cycle, the first testing day (blood tests) for all females was individually scheduled to take place during the mid-follicular phase (5–10 days after the last period) and the second testing day (vascular examination), as aforementioned above, was performed within 1 week after blood testing. Participants were also asked to give the date of their next period to have accurate information about the menstrual cycle phases of each studied women in regard to the time of blood and vascular testing. Based on this information, it appeared that blood tests occurred in both studied group of women in the mid-follicular phase (6th day of the cycle in CON and 7th day in ATH) and vascular tests in the ovulatory phase of the menstrual cycle (18th day of the cycle in CON and 14th day in ATH). The mean menstrual cycle length was 30.4 ± 3.9 days in CON and 28.8 ± 2.2 days in ATH.

### Anthropometric measurements

Anthropometric measurements were performed in the morning during the first testing day, about 15 min before blood was drawn. Each female participant's body mass and body height were measured using standard procedures and equipment (Radwag WPT 150, Radom, Poland), and body fat percentage was determined using bioelectrical impedance analysis (TANITA UM-018 Europe GmbH, Sindelfingen, Germany).

### Blood collection

Blood for serum biochemistry (sodium—Na^+^, potassium—K^+^, creatinine—Cr, albumin—Alb, ferritin—FER, c-reactive protein—CRP, alpha-1-acid glycoprotein—AAG, total creatine kinase—CK, glucose—GLU, hyaluronic acid—HA, syndecan-1—SDC-1, brain-derived neurotrophic factor—BDNF), lipid profile (total cholesterol—TC, low density lipoprotein—LDL, high density lipoprotein, HDL, triglyceride—TG) and hormone concentrations (testosterone—T, sex hormone-binding globulin—SHBG, cortisol—C, insulin—INS) was collected into plain tubes and left to clot for a minimum of 30 min at room temperature and then centrifuged at 1469×*g* for 10 min at 4 °C. Blood for plasma interleukin-6 (IL-6), tumor necrosis factor-alpha (TNF-α), sum of nitrite and nitrate (NO_x_), 6-keto-prostaglandin F1α (6-keto-PGF_1α_) and 1-methylnicotinamide (MNA) concentrations was collected in plain tubes containing EDTA and then centrifuged at 653×*g* for 15 min at 4 °C. Serum and plasma was stored at − 80 °C until analysis.

### Blood analysis

#### Blood morphology and biochemistry

Hemoglobin (Hb), hematocrit (Hct), erythrocyte (E), leukocyte (L) and platelet (PLT) count were analyzed by optical method with an Advia 2120 automated hematological analyzer (Siemens Healthcare Diagnostics, Tarrytown, NY, USA). Determination of serum Na^+^, K^+^, Cr, total CK and Alb concentrations were performed on a Roche Cobas c501 automated analyzer (Mannheim, Germany) using kits supplied by the manufacturer. TC, TG, LDL and HDL concentrations were determined in serum by enzymatic colorimetric method according to the manufacturer's protocol using the Cobas c501 analyzer (Roche Diagnostics, Mannheim, Germany). Serum FER was measured by chemiluminescence method using the Architect i1000SR analyzer (Abbott Laboratories, Chicago, IL, USA). CRP and AAG concentrations in serum were determined by the nephelometric method using Siemens reagents on a BN Prospect Siemens-Dade Behring analyzer (Marburg, Germany). Serum SDC-1 and HA concentrations as well as plasma IL-6, TNF-α, 6-keto-PGF_1α_ (a stable metabolite of prostacyclin) and BDNF were determined using a commercially available enzyme-linked immunosorbent assay kits (Diaclone Research, Besancon, France-SDC-1; Corgenix, Inc., Broomfield, USA-HA; R&D Systems, Inc. Minneapolis, USA-IL-6 and TNF-α; Enzo Life Sciences, NY, USA-6-keto-PGF_1α_; Promega Corp., Madison, USA-BDNF). All tests were conducted according to manufacturer's instructions. Intensity of the colorful reaction was estimated in optical density (OD) using the microplate reader (BioTek Instruments, Winooski, VT, USA) at 450 nm (SDC-1, HA, TNF-α and BDNF), 490 nm (IL-6) and 405 nm (6-keto-PGF_1α_). Concentration of plasma MNA was determined using the LC/MS/MS method as described earlier^26^. Briefly, chromatographic analysis was performed on an UltiMate 3000 HPLC system (Thermo Scientific Dionex, Sunnyvale, CA, USA) and chromatographic separation was carried out on an Aquasil C18 analytical column (4.6 mm × 150 mm, 5 μm, Thermo Scientific, Waltham, MA, USA). Detection was performed on a TSQ Quantum Ultra triple quadrupole mass spectrometer (Thermo Scientific) equipped with a heated electrospray ionization interface (HESI II Probe) operating in the positive ion mode. Data acquisition and processing were accomplished using Xcalibur 2.1 software^26^. The nitrate and nitrite concentration in plasma was measured by sensitive high-pressure liquid chromatography techniques (ENO-20 NO_x_ Analyzer; EiCom, Kyoto, Japan) and in this paper a nitrate and nitrite concentrations are summed and abbreviated to NO_x_.

#### Hormone measurements

Serum T, C, SHBG and INS concentrations were determined by electrochemiluminescence immunoassay using the Cobas e411 analyzer (Roche Diagnostics, Mannheim, Germany). The detection limits were 0.09 nmol L^−1^, 1.00 nmol L^−1^, 0.80 nmol L^−1^ and 1.39 pmol L^−1^, respectively for T, C, SHBG and INS. The intra and interassay CV for these measurements were 3.4% and 5.9%, 1.2% and 1.6%, 2.4% and 3.7%, 1.9% and 2.6%, respectively for T, C, SHBG and INS.

#### Calculations

In order to present non-HDL concentration, HDL cholesterol concentration was subtracted from a TC concentration. We have also calculated the homeostatic model assessment of insulin resistance (HOMA-IR) as follows: HOMA-IR = [Insulin (lU/mL) × Glucose (mmol/L)]/22.5. Moreover, free testosterone (fT) was calculated using the assumption-free empirical equations^27^.

### Endothelial function and arterial wall stiffness assessment

After arriving to the laboratory, the subjects rested for 20 min in the supine position in a quiet, semi-darkened and temperature-controlled (22–25 °C) room. During the tests, heart rate was monitored continuously using a three-lead electrocardiogram (ECG) and a 14 × 50 cm automatic cuff (Endothelix, Vendys, Palo Alto, CA, USA) was placed around the right forearm. Central systolic (cSP) and diastolic blood pressure (cDP) were measured noninvasively using the SphygmoCor system (AtCor Medical Pty Ltd, West Ryde, Australia). Cenrtal pulse pressure (cPP) was calculated as a difference between cSP and cBP.

Flow-mediated dilation (FMD), pulse wave velocity (PWV), augmentation index (AI), stiffness index (SI) and carotid intima media thickness (cIMT) were determined to assess endothelial function and arterial wall stiffness in non-invasive manner as it was described in our previous paper^18^. Briefly, FMD of the brachial artery (scanned above the antecubital fossa in the longitudinal plane) was performed based on the images provided by a linear-array ultrasound transducer (Acuson S2000, Siemens, Erlangen, Germany), while the occlusion cuff was placed on the forearm. Percent changes in flow-mediated brachial artery dilation after 5 min occlusion and cuff deflation were obtained by means of a custom made wall-tracking computer system and the algorithm for tracking the borders of the arterial walls was based on the active contour method. In case of pulse wave velocity (PWV), it was determined at the carotid and radial arterial sites with SphygmoCor system (AtCor Medical Pty Ltd, West Ryde, Australia) that uses applanation tonometry in conjunction with a 3-lead ECG to take sequential measurements at two arterial sites and pulse wave analysis was performed with the calculation of arterial stiffness parameters (central augmentation index, cAI). Carotid-radial PWV was calculated as the distance difference between the radial and carotid artery divided by the difference between transit time of the pulse wave from the left ventricle to the carotid artery (t1) and from the left ventricle to the radial artery (t2). On the other hand, cAI was defined as a percentage of the central pulse pressure attributed to the reflected pulse wave. To determine the stiffness index (SI), a PulseTrace photoplethysmographic device was used (CareFusion, Basingstoke, UK) and the measurement was based on the digital volume pulse measurements. SI was defined as the subject body weight divided by the peak to peak time of systolic and diastolic components of the pulse wave. At the end, the carotid intima-media thickness (cIMT) was measured using high-resolution B-mode ultrasonography (Acuson S2000, Siemens, Erlangen, Germany). Each side common carotid artery image from longitudinal postero- and anterolateral scans were obtained and cIMT was measured in the near and far walls in the most thickened area of each vessel. The Syngo Arterial Health Package (Siemens) software was used to process B-mode images and quantify cIMT.

### Statistical analysis

Comparisons between CON and ATH group were performed using the t-test for independent samples or Mann–Whitney U test if the assumptions for parametric analyses were violated. The normality of data distribution was tested with the Shapiro–Wilk test. The correlations between average amount of exercise training and androgen status as well as markers of endothelial function were evaluated using the Spearman’s *rho* correlation coefficient because of non-normal distribution of the exercise training (h/week) data. On the other hand, the correlations between anabolic-catabolic hormone balance indices (T/C and fT/C ratio) and endothelial function markers (FMD and HA) were evaluated using Pearson’s *r* or Spearman’s *rho* correlation coefficient if the assumption were not met. The data points that deviated from the group means by more than 3 SDs were treated as outliers and excluded from further analysis. The results presented in this study are expressed as means and standard deviation (SD) and the significance level was set at *p* < 0.05. The analyses were performed using TIBCO Software Inc. (2017), Statistica (data analysis software system), version 13, http://statistica.io.

### Ethical approval

The study protocol was conducted in accordance with the Declaration of Helsinki for research on human subjects and ethical approval for experimental procedure was obtained from the Local Ethical Committee at the Regional Medical Chamber in Krakow, Poland (opinion no. 48/KBL/OIL/2009).

### Consent to participate

All female volunteers gave written informed consent to participate in this study and to publish their research data anonymously in scientific journals.

## Results

### Physical characteristics and basic cardiovascular, blood morphology and biochemistry parameters

There were minor differences in basic anthropometric, cardiovascular, hematological and serum biochemical parameters between controls and female athletes (see Table 1). ATH were on average taller (+ 4.5 cm, 3% difference), they had significantly lower resting heart rate (− 17 bt min^−1^, 27% difference) and higher basal serum creatinine concentration (+ 8.7 μmol L^−1^, 12% difference). The Cr concentration was well within the clinical range in controls as well as in female athletes.

### Cardiometabolic health biomarkers

Table 2 presents the cardiometabolic health profile of the athletes and control group, including blood lipid profile, glucose homeostasis markers, inflammatory markers, arterial wall characteristics and level of vasoactive mediators. Although, there are no profound differences between these two groups of young women in these respects, slightly better cardiometabolic health status was seen in athletes. Particularly, they had better glucose control inferred from significantly lower basal serum Glu and INS concentrations and lower level of HOMA-IR (60% difference), indicating lower insulin resistance. Moreover, the female athletes had significantly higher serum HDL and lower AAG concentrations. Interestingly, they also presented with significantly enhanced basal serum MNA and BDNF concentrations when compared to control group (54 and 27% difference, respectively). Only one disadvantageous change was found in athlete women—namely, the significantly higher CK concentration, suggestive for muscle damages (in front of no adverse changes in other inflammatory markers) as a result of heavy training stress. No significant differences between female athletes and control group were demonstrated for any arterial wall parameter (see Table 2).

**Table 2:** Basal blood lipid profile, glucose homeostasis markers, inflammatory markers and arterial vasoactive mediators as well as arterial wall characteristics in controls (CON, n = 15) *vs* female athletes (ATH, n = 15).

	CON	ATHLETES	*p *value
Lipid profile			
TC, mmol L^−1^	4.01 ± 0.56	4.34 ± 0.55	0.11
LDL, mmol L^−1^	2.23 ± 0.54	2.21 ± 0.54	0.91
HDL, mmol L^−1^	1.66 ± 0.24	1.87 ± 0.29	0.04
Non-HDL, mmol L^−1^	2.35 ± 0.54	2.48 ± 0.62	0.56
TG, mmol L^−1^	0.78 ± 0.21	0.84 ± 0.44	0.93
Glucose homeostasis markers			
Glu, mmol L^−1^	5.00 ± 0.29	4.70 ± 0.29	0.01
INS, pmol L^−1^	51.9 ± 20.1	28.9 ± 8.2	0.003
HOMA-IR	1.94 ± 0.81	1.05 ± 0.39	0.003
Inflammatory markers			
FER, µg L^−1^	22.83 ± 9.85	24.23 ± 13.81	1.0
CRP, mg L^−1^	0.44 ± 0.32	0.55 ± 0.48	0.89
AAG, g L^−1^	0.76 ± 0.12	0.64 ± 0.16	0.002
IL-6, ng L^−1^	1.61 ± 1.39	1.18 ± 0.92	0.59
TNF-α, ng L^−1^	1.03 ± 0.73	0.86 ± 0.35	1.0
CK, IU/L	85.1 ± 25.0	152.8 ± 50.8	0.0007
Arterial wall characteristics			
cAI, %	2.53 ± 10.94	4.27 ± 10.20	0.65
PWV, m s^−1^	7.45 ± 0.97	7.51 ± 0.57	0.87
SI, m s^−1^	6.06 ± 0.92	5.67 ± 0.49	0.27
cIMT, mm	0.422 ± 0.037	0.443 ± 0.036	0.14
Vasoactive mediators			
NO_x_, µg L^−1^	29.92 ± 8.97	27.29 ± 11.48	0.25
6-Keto-PGF_1α_, ng L^−1^	5910 ± 2209	4977 ± 1634	0.19
MNA, µmol L^−1^	0.098 ± 0.034	0.170 ± 0.110	0.03
BDNF, ng L^−1^	31,323 ± 13,123	41,083 ± 12,540	0.045

Data are given as mean ± SD. *TC* total cholesterol, *LDL* low density lipoprotein, *HDL* high density lipoprotein, *non-HDL* non-high density lipoprotein, *TG* triglycerides, *Glu* glucose, *INS* insulin, *HOMA-IR* homeostatic model assessment, *FER* ferritin, *CRP* c-reactive protein, *AAG* alpha-1-acid glycoprotein, *IL-6* interleukin-6, *TNF-α* tumor necrosis factor-alpha, *CK* total creatine kinase, *cAI* central augmentation index, *PWV* pulse wave velocity, *SI* stiffness index, *cIMT* carotid intima-media thickness, *NO*_*x*_ the sum of nitrate and nitrite, *6-keto-PGF*_*1α*_ 6-keto-prostaglandin F_1α_, *MNA* 1-methylnicotinamide, *BDNF* brain-derived neurotrophic factor.

### Androgen status

It was demonstrated that basal serum T and fT concentrations were significantly lower (36 and 44% difference, respectively) and basal serum C concentration significantly higher (21% difference) in female athletes (see Fig. 1a–c). As a result, T/C and fT/C ratios were significantly lower (53 and 67% difference, respectively) in this group when compared to controls (Fig. 1d–e).

### Endothelial function

**Figure 2:**
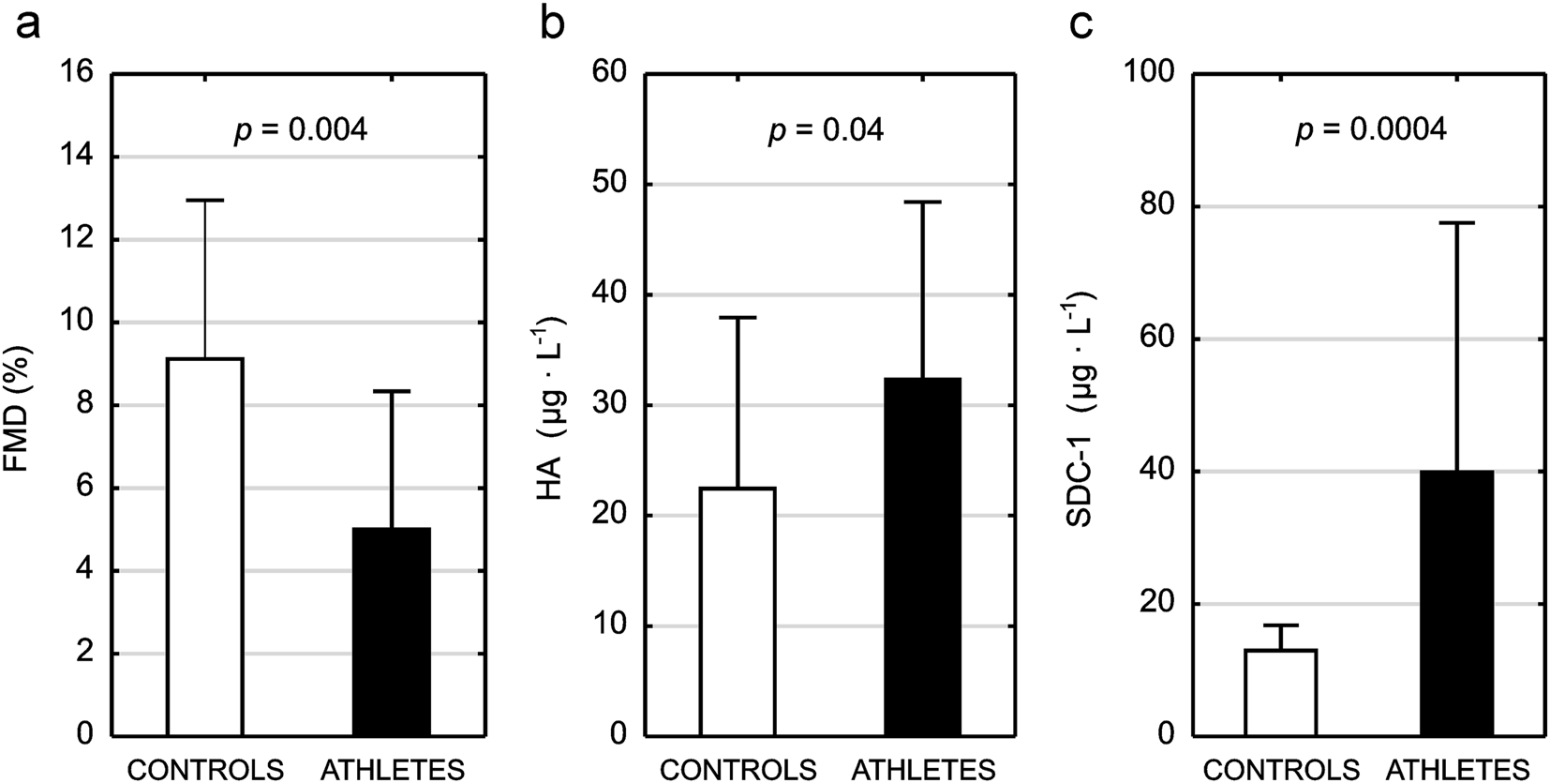
Endothelial function and glycocalyx layer integrity markers in female athletes and age-matched control group. Flow-mediated dilation (FMD—panel **a**); hyaluronic acid (HA—panel **b**), syndecan-1 (SDC-1—panel **c**).

FMD was markedly and significantly lower (58% difference, Fig. 2a), whereas HA and SDC-1 were significantly higher in female athletes when compared to controls (36 and 102% difference, respectively, see Fig. 2b,c).

### Correlations

#### Correlations between training load and androgen status, biochemical markers as well as endothelial function

Table 3 demonstrates bivariate correlations between the average amount of the exercise training and androgen status, biochemical markers as well as endothelial function for all studied women. It was found that the anabolic-catabolic hormone balance (T/C and fT/C ratio), AAG and FMD were significantly inversely correlated with the average time spent on exercise training per week. Conversely, basal serum C, CK, HA and SDC-1 concentrations were significantly positively correlated with the average amount of exercise training (Table 3).

**Table 3:** Bivariate correlations between training load (expressed as average amount of exercise training in hours per week) and androgen status, biochemical markers as well as endothelial function for all studied subjects (n = 30).

	Exercise training h/week	
	Spearman’s *rho*	*p* value
T, nmol L^−1^	− 0.25	0.17
SHBG, nmol L^−1^	0.28	0.14
fT, pmol L^−1^	− 0.28	0.15
C, nmol L^−1^	0.44	0.02
T/C, × 10^3^	− 0.59	0.001
fT/C, × 10^3^	− 0.53	0.004
CK, IU/L	0.61	0.0005
AAG, g L^−1^	− 0.38	0.04
FMD, %	− 0.47	0.008
HA, µg L^−1^	0.36	0.05
SDC-1, µg L^−1^	0.53	0.003

Spearman’s *rho* is given due to non-normal distribution of the exercise training (h/week) data. *T *testosterone, *SHBG* sex hormone-binding globulin, *fT* free testosterone, *C* cortisol, *T/C* testosterone to cortisol ratio, *fT/C* free testosterone to cortisol ratio, *CK* total creatine kinase, *AAG* alpha-1-acid glycoprotein, *FMD* flow-mediated dilation, *HA* hyaluronic acid, *SDC-1* syndecan-1.

#### Correlations between anabolic-catabolic hormone balance and endothelial function

**Figure 3:**
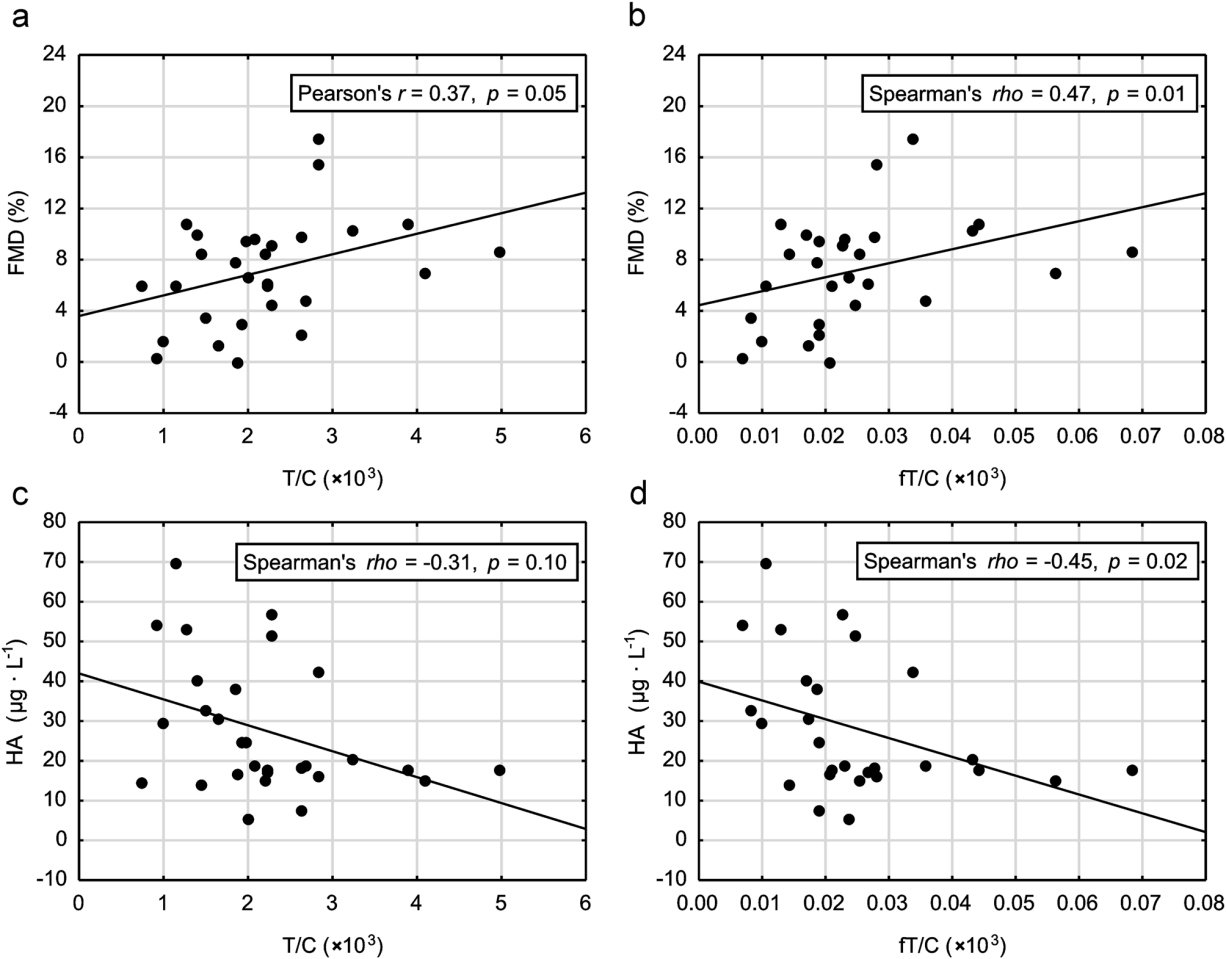
Correlations between androgen status (testosterone to cortisol ratio—T/C and free testosterone to cortisol ratio—fT/C) and flow-mediated dilation (FMD), panel (**a**,**b**) and between androgen status (T/C and fT/C) and hyaluronic acid (HA), panel (**c**,**d**), in all studied women (n = 30).

The anabolic-catabolic hormone balance (T/C and fT/C ratio) was significantly correlated with endothelial markers—positively with FMD and negatively with basal serum HA concentration (see Fig. 3a–d).

## Discussion

The main and original finding of this study was: (1) female athletes presented with lower FMD and higher basal concentrations of HA and SDC-1 when compared to control group (Fig. 2a–c); (2) attenuation of endothelial function was accompanied by significantly lower T and fT concentrations, higher C concentration and as a result, significantly lower T/C and fT/C ratios in athletes than in controls (Fig. 1a–e), and (3) T/C and fT/C ratios were significantly positively correlated with FMD and negatively with HA in all studied women (Fig. 3a–d). Female athletes had also 2-folds higher basal concentration of CK illustrating a greater “stress” exerted on their skeletal muscles as well as significantly lower concentration of AAG, considered as a candidate marker of overtraining^28^ (see Table 2), but did not display increased inflammatory response as based on basal blood IL-6, TNF-α and CRP levels. We postulate that worsened endothelial function, together with increased glycocalyx shedding but without a robust systemic inflammatory response may be a feature of high training stress that could be potentially related to overreaching and overtraining syndrome. To our knowledge, this is the first study showing the deterioration of anabolic–catabolic hormonal balance together with endothelial function as a result of physical training in athletes. Simultaneously, we are not aware of any data demonstrating that endothelial dysfunction (low or decrease in FMD and increased glycocalyx shedding) can be considered as a tool to monitor athletes’ vascular health-risk in the time-course of training.

### FMD responses to training

The decrease in FMD in response to exercise training may seem unexpected since the opposite effect has been reported from early studies on training-induced changes in endothelial function^29^. The recent available data also show improved FMD after the training intervention both in healthy^22,30–32^ and diseased subjects^33,34^. Additionally, some of these studies revealed that the high intensity interval training regimen (HIIT) is more effective in increasing FMD than moderate intensity continuous training (MICT)^30–32^. Such conclusion was also drawn in the meta-analysis by Ramos et al.^35^ who reported that HIIT is more potent stimulus than MICT in enhancing vascular function. Taking together, it appears that high intensity training have beneficial effects on FMD, but at the same time, we do not think that this fact contradicts our results (showing deterioration in FMD after physical training in athletes).

It has to be stressed out that professional training program performed by athletes at national level differs drastically from HIIT regimens used in experimental studies. As it was described in Methods section, total training load in ATH group amounted on average to ~ 13 h per week, whereas HIIT session lasts usually no longer than 30 min, repeated on average 3 times per week and only about half of the time of HIIT session is devoted to exercise in the heavy-intensity domain (see e.g.^35^). This results in about 1.5 h of training per week in total what is almost 9 times less as compared to training load experienced by our female athletes. From this perspective, it is not surprising that in this study we reported a different response from that observed after heavy intensity interval training programs. It may be suggested that high-intensity training of low volume is able to induce beneficial effects on vascular function (at least on short-term) and leads to increase in FMD, but moderate-to-high intensity training of high volume may lead to opposite responses and decrease in FMD. Therefore, the time-point of the FMD measurement in regard to the training loads performed by the athletes may at least partly explain the differences reported in previous studies as regards FMD values in response to athletic training^17–21^. Of note, in our previous study^18^, we have found no differences in brachial artery FMD between young male athletes competing at national and international level and the controls. The athletes were tested in the above mentioned investigation in the most suitable time for evaluation the effect of long-term athletic training on basal vascular function, i.e. after the transition phase of annual training program, when the training loads were greatly reduced. In our view, it contributed to the lack of differences in FMD between the athletes and nonathletes. However, it should be mentioned that decreased FMD in athletic population may occur as a result of structural remodeling of the artery and decrease in wall-to-lumen ratio (see^17^), but this does not seem to be the case in our study. We have found that there were no differences in the arterial wall characteristics between our female athletes and controls (see Table 2), which indicates—together with enhanced markers of glycocalyx degradation in ATH—on endothelial dysfunction as the main reason for reduced FMD.

These findings may be supported by the experiments in which the decreased FMD in amenorrheic athletes^36,37^ or in female athletes with negative energy availability^38^ was observed. Although, the etiology of the athletic amenorrhea is complex, it is widely accepted that it is developed as a consequence of low energy availability related to excessive training loads^39^. Already in 1990, Keizer and Rogol^40^ put forward the hypothesis that athletes with a training-induced menstrual cycle alterations are overreached or overtrained. Taking these into account, it seems reasonable to expect that heavy training loads resulting in high oxidative stress, inflammatory response, metabolic disturbances and disruption of hormonal regulation can lead to vascular dysfunction that was observed in the previous studies of amenorrheic women^36–38^. In our present work, the physical training performed by female athletes impaired hormonal balance (Fig. 1a–e) and endothelial function (Fig. 2a–c) without changes in metabolic and inflammatory status (see Table 2). We postulate that such changes may be an early sign of the high training stress experienced by athletes.

### Androgens and endothelium

Here, we have demonstrated that female athletes were characterized by significantly lower androgen status (lower T, fT, T/C, fT/C and higher C level) that was often used as an indication of the training-induced fatigue and overload^4,41^ or even overtraining^3^. The significant positive correlations between FMD and androgen status (T/C and fT/C ratios—Fig. 3a,b) suggest on one hand that decreased FMD may be a valuable indicator of excessive training stress, and on the other, that attenuated androgens and endothelial disturbances occurring in response to training stress are interrelated. This assumption seems to be supported by the significantly higher concentrations of HA and SDC-1 in trained females (Fig. 2b,c) and by the negative correlations between T/C and fT/C and hyaluronan concentration (Fig. 3c,d). Moreover, the exercise training load was negatively correlated with androgen status and FMD and positively correlated with the concentrations of HA and SDC-1 in all women studied (Table 3). These results also support the concept that the higher the training load, the lower the androgen status and FMD, and the higher the levels of endothelial glycocalyx degradation markers.

Low androgen levels are associated with adverse effects on cardiovascular system (particularly on endothelium) in both men and women (see^42^). Usually, this negative effect was linked to androgen-related regulation of the inflammatory process^6,43^, but it was also postulated that androgens may exert a direct actions on vascular endothelium in humans (see^44,45^). It was demonstrated that androgens lead to endothelial nitric oxide synthase (eNOS) activation^44,46^, therefore the decreased androgen concentrations in ATH women may be responsible for lowered NO^•^ synthesis and observed reduction in FMD. The lack of significant differences in plasma NO_x_ concentration does not exclude this conclusion, as in many cases systemic endothelial dysfunction did not lead to low levels of NO_x_ due to the activation of reductive pathway of NO^**·**^ synthesis^47^ and NO_x_ was also not impaired after exercise despite impaired endothelium-dependent vasodilation^11^.

At this point, it should be also noted that in this study we did not measure ovarian hormone levels and we cannot completely rule out a potential role of estradiol on FMD, even when we put the effort to control as much as possible the same timing of both blood and vascular testing during the menstrual cycle (see “Methods”). At the same time, however, it should be taken into account that the data on the significant effect of menstrual cycle phase on endothelial function are conflicting. Although, it seems reasonable that low-estrogen phase of the menstrual cycle could be different from high-estrogen phase^48^, a number of studies did not find significant effect of the menstrual cycle on endothelial function (see e.g.^49–52^). Additionally, any potential effect of menstrual cycle phase on endothelial function seems to be insufficient to explain 58% difference in FMD value between controls and female athletes observed in this study (see^53^).

### Endothelial function and athletic training

The deterioration of endothelial function in ATH group appears to be also related to shedding of the glycocalyx, because the significantly higher basal concentrations of HA and SDC-1 were noticed in athletes (Fig. 2b,c). Of interest, in our previous study with 20-week long controlled moderate intensity endurance training^54^, it was shown for the first time that the applied training program in the previously untrained subjects led to a decrease in basal SDC-1 and heparan sulfate (HS) concentrations what was accompanied by attenuation of oxidative stress and enhancement of antioxidant defense. These opposite findings indicate that training-induced effect on the endothelial glycocalyx integrity is dependent on the applied training loads and in trained individuals might vary depending on the training period. In the above mentioned training study^54^, the exercise intensity was mostly moderate and the total training time was 2 h and 40 min per week—it cannot be compared with the training loads experienced by the ATH group from this investigation (on average ~ 13 h per week). The training response seems to be similar to exercise-induced acute changes in the markers of glycocalyx shedding, because it was shown that sessions with shorter duration of heavy or maximal intensity exercise does not negatively affect glycocalyx integrity^55^, but prolonged exposure to heavy-intensity exercise can lead to the shedding of glycocalyx components^10,56,57^. It was argued that an increase in markers of glycocalyx shedding—mainly SDC-1, HS and HA—occurred as a result of enhanced blood flow^56^, decrease in antioxidative capacity^57^ and increase in reactive oxygen species (ROS) and cytokines^10^.

In our view, all these proposed mechanisms can act in concert, leading to high oxidative stress that was recently experimentally shown to induce degradation of endothelial glycocalyx through augmented histone deacetylase (HDAC) activity that upregulates matrix metalloproteinases (MMPs) and downregulates their inhibitors (TIMPs—tissue inhibitors of metalloproteinase)^58^. These findings correspond to the exercise training studies demonstrating that high intensity exercise enhances serum MMP-1^59,60^ that was shown to cleave SDC-1 (see^61^). Moreover, the basal serum level of MMP-1 was increased in response to resistance training^59^ that is also an important part of the preparatory period phase of the season in track and field athletes. At the end, it could be hypothesized that low androgen levels are also involved in glycocalyx shedding through their effect on unbalanced MMP enzymatic degradation of the extracellular matrix (see^62^), but this finding certainly needs further investigation.

### MNA and BDNF in endothelial function

Whatever the mechanism for endothelial dysfunction, it seems that resting brachial artery FMD measurement can be considered as a tool to monitor athletes’ health-risk in the time-course of training. It is interesting that lower FMD, accompanied by higher HA and SDC-1 concentrations in our female athletes was observed despite normal cardiometabolic and inflammatory profile (see Table 2). What is more, athletes have demonstrated improved blood glucose homeostasis (lower basal Glu, INS and HOMA-IR) and higher basal concentrations of HDL, BDNF and MNA (Table 2)—changes that are associated with training adaptation (see e.g.^18,63,64^). This indicates that cardiometabolic and inflammatory markers are less sensitive to potential training stress than endothelial function and anabolic–catabolic hormonal markers. The increased MNA and BDNF level may be considered as a sign of adaptive vascular response to the training triggered to mitigate decreased NO bioavailability, since it was reported that both molecules exerts vasoprotective effects through prostacyclin release^65,66^. Moreover, in case of the BDNF level, we have found no difference in the PLT count between the athletes and the controls which indicates that the higher level of BDNF in female athletes was not attributable to higher release from the platelets—the main source of serum BDNF^67,68^.

### Limitations

Despite of presenting these new findings, we are aware of the main limitation of this study, pertaining to cross-sectional design and single time-point measurement that could not provide a complete pre- and post-training characteristics of the studied females athletes. Nevertheless, our results clearly indicate that the impaired endothelial function (FMD, SDC-1, HA), accompanied by biomarkers of vascular adaptive response (MNA, BDNF), several biochemical (CK) and hormonal markers (T/C, fT/C) of the training stress, as shown in the present study, should receive more attention in the future in the context of overtraining and vascular health-risk in the training process in athletes. In addition, we did not measure ovarian hormone levels or check temperature or any other indicators of the menstrual cycle. Therefore, we cannot completely rule out a potential role of estradiol on endothelial function, even when we put the effort to control as much as possible the same timing of both blood and vascular testing during the menstrual cycle (see “Methods”).

### Conclusions

Summing up, we have demonstrated that female athletes developed endothelial dysfunction as evidenced by impaired endothelial-dependent dilation (FMD) and glycocalyx degradation that occurred together with anabolic–catabolic hormone balance disturbances. Our results clearly show that some kinds of exercise training in athletes may lead to adverse changes in hormonal and endothelial status that may have a long-term adverse vascular effects. Thus, our results call for a regular endothelial profiling in athletes undergoing physical training to reliably assess adaptive–maladaptive endothelial response to monitor the balance of adaptive/vasoprotective and maladaptive/detrimental effects of exhaustive training programs, to foster the earlier and to limit the later. This could reduce the cardiovascular-related health problems in athletes.

## Data Availability

The datasets generated during and analyzed during the current study are available from the corresponding author on reasonable request.
